# Supplementary material for: Low serum brain-derived neurotrophic factor may predict poor response to cardiac rehabilitation in patients with cardiovascular disease
Source: PLoS One. 2024 Feb 6;19(2):e0298223. doi: 10.1371/journal.pone.0298223 (PMC10846715; doi:10.1371/journal.pone.0298223)
Supplement: Attachment — Submitted filename: renamed_d531e.docx [file pone.0298223.s002.docx]

PONE-D-23-00760

Low serum Brain-Derived Neurotrophic Factor may predict poor response to cardiac rehabilitation in patients with cardiovascular disease

PLOS ONE

Dear Dr. Tsukada,

Thank you for submitting your manuscript to PLOS ONE. After careful consideration, we feel that it has merit but does not fully meet PLOS ONE’s publication criteria as it currently stands. Therefore, we invite you to submit a revised version of the manuscript that addresses the points raised during the review process.

Please submit your revised manuscript by Mar 24 2023 11:59PM. If you will need more time than this to complete your revisions, please reply to this message or contact the journal office at [plosone@plos.org](mailto:plosone@plos.org). When you're ready to submit your revision, log on to https://www.editorialmanager.com/pone/ and select the 'Submissions Needing Revision' folder to locate your manuscript file.

Please include the following items when submitting your revised manuscript:

- A rebuttal letter that responds to each point raised by the academic editor and reviewer(s). You should upload this letter as a separate file labeled 'Response to Reviewers'.
- A marked-up copy of your manuscript that highlights changes made to the original version. You should upload this as a separate file labeled 'Revised Manuscript with Track Changes'.
- An unmarked version of your revised paper without tracked changes. You should upload this as a separate file labeled 'Manuscript'.

If you would like to make changes to your financial disclosure, please include your updated statement in your cover letter. Guidelines for resubmitting your figure files are available below the reviewer comments at the end of this letter.

If applicable, we recommend that you deposit your laboratory protocols in protocols.io to enhance the reproducibility of your results. Protocols.io assigns your protocol its own identifier (DOI) so that it can be cited independently in the future. For instructions see: <https://journals.plos.org/plosone/s/submission-guidelines#loc-laboratory-protocols>. Additionally, PLOS ONE offers an option for publishing peer-reviewed Lab Protocol articles, which describe protocols hosted on protocols.io. Read more information on sharing protocols at <https://plos.org/protocols?utm_medium=editorial-email&utm_source=authorletters&utm_campaign=protocols>.

We look forward to receiving your revised manuscript.

Kind regards,

Rabbanie Tariq Wani
Academic Editor
PLOS ONE

Journal Requirements:

When submitting your revision, we need you to address these additional requirements.

1. Please ensure that your manuscript meets PLOS ONE's style requirements, including those for file naming. The PLOS ONE style templates can be found at
   https://journals.plos.org/plosone/s/file?id=wjVg/PLOSOne_formatting_sample_main_body.pdf and 
   https://journals.plos.org/plosone/s/file?id=ba62/PLOSOne_formatting_sample_title_authors_affiliations.pdf

**Response:**

We have corrected our manuscript to meet the style requirements of *PLOS ONE*.

**2. We noticed you have some minor occurrence of overlapping text with the following previous publication(s), which needs to be addressed:**

**- https://doi.org/10.1093/eurheartj/ehy565.P2552**

In your revision ensure you cite all your sources (including your own works), and quote or rephrase any duplicated text outside the methods section. Further consideration is dependent on these concerns being addressed.

**Response:**

Thank you for this comment. We are afraid that you may have mistakenly provided the doi (https://doi.org/10.1093/eurheartj/ehy565.P2552) of an abstract of ESC2018 meeting: P2552 “Cardiac rehabilitation revisited: time to change standard practices?” by De La Porte et al. There is no overlap in the text between our manuscript and the abstract by De La Porte et al. The doi that you intended may have been https://doi.org/10.1093/eurheartj/ehy565.P2551, an abstract of our presentation at the poster session of ESC2018: P2551 “Low serum brain-derived neurotropic factor may predict poor response to cardiac rehabilitation in patients with cardiovascular disease”.

Based on your comment and this assumption, we have rewritten the Methods section of the revised manuscript to avoid any overlap in text, as follows:

***Page 2, Methods section of the Abstract***

“**Methods:** We assigned 99 CVD patients (mean age, 71±12 years; 60 males) to Low, Middle, and High groups based on tertiles of baseline BDNF level. Cardiopulmonary exercise testing was performed using supervised bicycle ergometer twice, before and after 3 weeks of CR.”

3. In the ethics statement in the Methods and online submission information, please ensure that you have specified (1) whether consent was informed and (2) what type you obtained (for instance, written or verbal, and if verbal, how it was documented and witnessed). If your study included minors, state whether you obtained consent from parents or guardians. If the need for consent was waived by the ethics committee, please include this information.
If you are reporting a retrospective study of medical records or archived samples, please ensure that you have discussed whether all data were fully anonymized before you accessed them and/or whether the IRB or ethics committee waived the requirement for informed consent. If patients provided informed written consent to have data from their medical records used in research, please include this information.

**Response:**

In accordance with your request, we have rewritten the original sentence to read, “Informed consent was obtained from all patients,”. Also, we apologize for mistakenly providing an incorrect approval number (18291); this has also been corrected in the revised version of the manuscript, as follows:

***Study Patients subsection of the Methods (page 5, lines 72–73)***

“Written informed consent was obtained from all patients, and the study was approved by the ethics committee at Kurume University (approval no. 18391).”

4.  Please review your reference list to ensure that it is complete and correct. If you have cited papers that have been retracted, please include the rationale for doing so in the manuscript text, or remove these references and replace them with relevant current references. Any changes to the reference list should be mentioned in the rebuttal letter that accompanies your revised manuscript. If you need to cite a retracted article, indicate the article’s retracted status in the References list and also include a citation and full reference for the retraction notice.

**Response:**

No particular changes have been made.

**Additional Editor Comments:**
One of the reviewers have suggested for a minor review, please find there comments attached

[Note: HTML markup is below. Please do not edit.]

Reviewers' comments:

Reviewer's Responses to Questions

Comments to the Author

1. Is the manuscript technically sound, and do the data support the conclusions?

The manuscript must describe a technically sound piece of scientific research with data that supports the conclusions. Experiments must have been conducted rigorously, with appropriate controls, replication, and sample sizes. The conclusions must be drawn appropriately based on the data presented.

Reviewer #1: Yes

2. Has the statistical analysis been performed appropriately and rigorously?

Reviewer #1: Yes

3. Have the authors made all data underlying the findings in their manuscript fully available?

The [PLOS Data policy](http://www.plosone.org/static/policies.action#sharing) requires authors to make all data underlying the findings described in their manuscript fully available without restriction, with rare exception (please refer to the Data Availability Statement in the manuscript PDF file). The data should be provided as part of the manuscript or its supporting information, or deposited to a public repository. For example, in addition to summary statistics, the data points behind means, medians and variance measures should be available. If there are restrictions on publicly sharing data—e.g. participant privacy or use of data from a third party—those must be specified.

Reviewer #1: Yes

4. Is the manuscript presented in an intelligible fashion and written in standard English?

PLOS ONE does not copyedit accepted manuscripts, so the language in submitted articles must be clear, correct, and unambiguous. Any typographical or grammatical errors should be corrected at revision, so please note any specific errors here.

Reviewer #1: Yes

5. Review Comments to the Author

Please use the space provided to explain your answers to the questions above. You may also include additional comments for the author, including concerns about dual publication, research ethics, or publication ethics. (Please upload your review as an attachment if it exceeds 20,000 characters)

Reviewer #1: The authors showed that serum BDNF levels can predict the effect of exercise therapy on patients with cardiovascular disease. Furthermore, they showed that serum BDNF levels were associated with patient nutritional status. This reviewer considers this study to be an interesting study. I have some questions.

1. Nakano et al. reported that serum BDNF levels were associated with skeletal muscle strength, but not skeletal muscle mass (Int Heart J 2020;61(1):96-102). Present study also showed no association between BDNF levels and skeletal muscle mass, consistent with previous result. What indicators of skeletal muscle strength are measured in this study?

**Response:**

Unfortunately, we have no data on skeletal muscle strength in this study. We have thus added the following sentences to the revised manuscript, to read:

***Discussion (page 16, lines 205-209)***

“Nakano et al. reported that serum BDNF levels in patients with heart failure were associated with skeletal muscle strength, but not with skeletal muscle mass [28]. Given that finding, the association between serum BDNF levels and skeletal muscle strength in CVD patients warrants attention. This issue should be addressed in future research.”

The new reference you so kindly provided has been added as Reference 28.

“28.	Nakano I, Kinugawa S, Hori H, *et al*. Serum brain-derived neurotrophic factor levels are associated with skeletal muscle function but not with muscle mass in patients with heart failure. Int Heart J 2020;61:96-102.”

2. Previous reports have shown that serum BDNF levels are decreased in patients with heart failure and that serum BDNF and peak oxygen uptake are closely related (J Card Fail 2015;21(4):300-306, Int J Cardiol 2013;168:e142-144). In this study, peak oxygen uptake before exercise therapy was not associated with serum BDNF levels. Please discuss these differences.

**Response:**

We greatly appreciate this pertinent comment. Discussion of this issue has been added to the revised manuscript, as follows:

***Discussion (page 15, line 193 to page 16, line 200)***

“Fukushima et al. showed a close association between serum BDNF and peak oxygen uptake in patients with heart failure [26,27]. In the present study, peak VO_2_ did not differ significantly among the Low, Middle, and High groups before exercise training (p=0.1113), although a marginal association was identified between baseline serum BDNF and peak VO_2_ in the total patient cohort (r=0.2116, p=0.0355). The reason for these differences between studies is unknown. One possible explanation is that more than half of the patients in the present study had CVD other than heart failure, whereas Fukushima et al. investigated the cohorts enrolling only patients with heart failure.”

The papers you mentioned have now been cited as References #26 and 27.

“26.	Fukushima A, Kinugawa S, Homma T, *et al*. Serum brain-derived neurotropic factor level predicts adverse clinical outcomes in patients with heart failure. J Card Fail 2015;21:300-306

27.	Fukushima A, Kinugawa S, Homma T, *et al*. Decreased serum brain-derived neurotrophic factor levels are correlated with exercise intolerance in patients with heart failure. Int J Cardiol 2013;168:e142-144.”

3. BDNF is believed to be secreted from skeletal muscle during exercise. Did serum BDNF levels change after exercise therapy?

**Response:**

We agree with the point you are making, but unfortunately we did not measure serum BDNF levels after exercise training. The following sentences have been added to the revised manuscript to address this:

***Discussion (page 18, lines 233 to page 19, line 235)***

“Next, it would be interesting to know whether serum BDNF changes after exercise treatment. Exercise-induced BDNF changes and the associations between BDNF and the effectiveness of CR should be investigated in future research.”

4. FIG. 1 shows the before-and-after changes of an individual patient. Therefore, please show the graph with a line connecting the points before and after so that you can see the change.

**Response:**

Thank you for this suggestion. We have added an appropriate indication of the intervention period in the revised version of Figure 1.

6. PLOS authors have the option to publish the peer review history of their article ([what does this mean?](https://journals.plos.org/plosone/s/editorial-and-peer-review-process#loc-peer-review-history)). If published, this will include your full peer review and any attached files.


If you choose “no”, your identity will remain anonymous but your review may still be made public.


Do you want your identity to be public for this peer review? For information about this choice, including consent withdrawal, please see our [Privacy Policy](https://www.plos.org/privacy-policy).

Reviewer #1: No

[NOTE: If reviewer comments were submitted as an attachment file, they will be attached to this email and accessible via the submission site. Please log into your account, locate the manuscript record, and check for the action link "View Attachments". If this link does not appear, there are no attachment files.]

While revising your submission, please upload your figure files to the Preflight Analysis and Conversion Engine (PACE) digital diagnostic tool, <https://pacev2.apexcovantage.com/>. PACE helps ensure that figures meet PLOS requirements. To use PACE, you must first register as a user. Registration is free. Then, login and navigate to the UPLOAD tab, where you will find detailed instructions on how to use the tool. If you encounter any issues or have any questions when using PACE, please email PLOS at [figures@plos.org](mailto:figures@plos.org). Please note that Supporting Information files do not need this step.

In compliance with data protection regulations, you may request that we remove your personal registration details at any time. [(Remove my information/details)](https://www.editorialmanager.com/pone/login.asp?a=r). Please contact the publication office if you have any questions.
