# Supplementary material for: Low serum brain-derived neurotrophic factor may predict poor response to cardiac rehabilitation in patients with cardiovascular disease
Source: PLoS One. 2024 Feb 6;19(2):e0298223. doi: 10.1371/journal.pone.0298223 (PMC10846715; doi:10.1371/journal.pone.0298223)
Supplement: Attachment — Submitted filename: Response to Editors .docx [file pone.0298223.s003.docx]

PONE-D-23-00760R1

Low serum brain-derived neurotrophic factor may predict poor response to cardiac rehabilitation in patients with cardiovascular disease PLOS ONE

Dear Dr. Tsukada,

Thank you for submitting your manuscript to PLOS ONE. One additional expert reviewer carefully evaluated your manuscript. The comments are included below. Although the reviewer and I find merit in your study, I myself found concerns about the statistical analysis that precludes its acceptance for publication in PLOS ONE.

I invite you to respond to the reviewer’s comments and my own, and submit a revision if you believe you can adequately address the concerns. Please note that this request for revision does not imply that the manuscript will ultimately be accepted, but that the manuscript must be meticulously revised before further consideration is provided.

Your revised submission must include an itemized, point-by-point response to the comments of the reviewer and editor.

Please submit your revised manuscript by Aug 19 2023 11:59PM. If you will need more time than this to complete your revisions, please reply to this message or contact the journal office at [plosone@plos.org](mailto:plosone@plos.org). When you're ready to submit your revision, log on to <https://www.editorialmanager.com/pone/> and select the 'Submissions Needing Revision' folder to locate your manuscript file.

Please include the following items when submitting your revised manuscript:

A rebuttal letter that responds to each point raised by the academic editor and reviewer(s). You should upload this letter as a separate ﬁle labeled 'Response to Reviewers'.

A marked-up copy of your manuscript that highlights changes made to the original version. You should upload this as a separate ﬁle labeled 'Revised Manuscript with Track Changes'.

An unmarked version of your revised paper without tracked changes. You should upload this as a separate ﬁle labeled 'Manuscript'.

If you would like to make changes to your financial disclosure, please include your updated statement in your cover letter. Guidelines for resubmitting your figure files are available below the reviewer comments at the end of this letter.

If applicable, we recommend that you deposit your laboratory protocols in [protocols.io](http://protocols.io/) to enhance the reproducibility of your results. Protocols.io assigns [your protocol its own identifier (DOI) so that it can be cited independently in the future. For instructions see: https://journals.plos.org/ plosone/s/submission-guidelines#loc-laboratory-protocols. Additionally, PLOS ONE offers an option for publishing peer-reviewed Lab](https://journals.plos.org/plosone/s/submission-guidelines#loc-laboratory-protocols) Protocol articles, [which describe protocols hosted on protocols.io. Read more information on sharing protocols at https://plos.org/protocols?utm_medium=editorial-email& utm_source=authorletters&utm_campaign=protocols.](https://plos.org/protocols?utm_medium=editorial-email&utm_source=authorletters&utm_campaign=protocols)

We look forward to receiving your revised manuscript. Kind regards,

Yuichiro Nishida Academic Editor PLOS ONE

Additional Editor Comments :

Major comments:

The topic of the present study is interesting, and the results are potentially important for the development of tailored, optimized exercise programs for patients with cardiovascular disease. However, I have concerns regarding the statistical analysis. To answer the main research question, you used paired t-tests to assess pre-post changes in AT and Peak VO2 within groups. This is not sufficient to answer the research question. Instead, you should statistically compare pre-post changes in AT and Peak VO2 “between” the three groups (instead of “within” groups). For instance, the changes (increases) in AT and Peak Vo2 in the high BDNF group are significantly greater than the changes (increases) in AT and Peak Vo2 in the low BDNF group? Additionally, to adjust for potential confounding factors, an analysis of covariance (ANCOVA) may be used to calculate the adjusted means (and 95% confidence intervals) of the changes in AT and Peak VO2 in the three groups. Although there were no statistically significant differences among the three groups for age, sex, main disease, comorbidities, medication, ejection fraction, or SMI at baseline, there may be tendencies that the subjects in the low BDNF group are older, have a lower percentage of males, a lower percentage of coronary artery disease, a higher percentage of post-operated, and a higher percentage of β blocker use (as shown in Table 1). These factors are considered potential confounding factors that should be adjusted as covariates in the multivariate analysis. Although you may not be familiar with multivariate analysis, you should use multivariate analysis (e.g., ANCOVA) to adjust for at least age and sex as covariates and statistically compare the adjusted means (and 95% confidence intervals) of the changes in AT and Peak VO2 “between” the three groups. According to the additional results obtained from the new analyses, the whole manuscript (especially, the methods section, the results section, and the discussion and conclusion sections) should be revised adequately.

***Authors’ Response***

Thank you for your comment. According to your suggestion, we performed ANCOVA followed by post-hoc analysis to compare age- and gender-adjusted means of the changes (%increase) in AT and peak VO_2_ among the three BDNF groups. As described below, 3-week CR-induced increase in peak VO_2_ was significantly lower in Low BDNF group than High BDNF group. New data were added and the following changes were done in the second revised (R2) version of our manuscript.

**Abstract**

Page 2/R2, Lines 37-Page 38/R2, Line 38

The following sentence was added, to read;

“Age- and gender-adjusted %increase in peak VO_2_ was significantly lower in Low group than High group (p<0.05).”

**Method section**

Page 9/2R, Lines 128-130

New sentence was added, as follows to read;

“Analysis of covariance (ANCOVA) followed by post-hoc analysis using Turkey’s HSD test was conducted to compare age- and gender-adjusted %increase in AT and peak VO_2_ after 3-week CR.”

**Results section**

Page 12/R2, Line 153-Page 13/R2, Line 156

The following sentences were added and new Figure 2 was created in the R2 manuscript.

“After adjustment with age and gender, %increase in peak VO_2_ after 3-week CR was significantly lower in Low BDNF group than in High BDNF group (P=0.0197) (Figure 2). However, age- and gender-adjusted %increase in AT did not differ among the three groups.”

**Discussion section**

Page 13/R2 Line 168-Page 14/R2 Line 170

New sentence was added as follows, to read;

“Age- and gender-adjusted %increase in peak VO_2_, but not AT, after 3-week CR was significantly lower in Low BDNF group than in High BDNF group.”

Page 15/R2, Lines 184-191

The original sentences (Page 14, Lines 169-177/R1) were rewritten as follows, to read;

“In the present study, the prevalence of these factors was similar among Low, Middle and High BDNF groups, as well as SMI, left ventricular ejection fraction, and the prevalence of heart failure, coronary artery disease, other cardiovascular diseases, the comorbidities, and medications. It is noteworthy that baseline AT and peak VO_2_, parameters of anaerobic capacity, did not differ among the three groups and that even after adjustment with age and gender, the CR-induced increase in peak VO_2_ was significantly lower in Low BDNF group than in High DBNF group. Thus, it is possible that low baseline BDNF may be an independent predictor of non-responders for CR in CVD patients.”

Minor comments:

1. How was the sample size (n = 99) determined?

***Authors’ Response***

We are sorry for our inadequate description regarding study patients. This retrospective cohort study included 99 patients, simply because they met the inclusion criteria: inhospital patients undergoing exercise stress test and serum BDNF sampling before and after 3 weeks of CR during the study period. Thus, the sample size was not predetermined. To clarify this point, the following changes were done in the R2 manuscript.

**Abstract**

Page 2/R2, Lines 28

The original words: “this study investigated…” (Page 2, Line 28/R1) was rewritten to “this study retrospectively investigated…”.

**Methods section**

Page 5/R2, Lines 69-71

The original sentence (Page 5, Lines 71-72/R1) was changed as follows, to read;

“We retrospectively studied 99 consecutive patients with CVD (age: 71±12, males 60) who were admitted to Kurume University Medical Center from February 2013 to April 2015 and underwent exercise stress test and serum BDNF sampling before and after 3 weeks of CR.”

2. How was the study participants’ adherence to the cardiac rehabilitation program in the three groups?

***Authors’ Response***

Thank you for your comment. The following sentence was added in the R2 manuscript, to read:

**Methods section**

Page 9/R2, Lines 119-120

“Adherence to the inpatient CR program was almost complete for all study subjects.”

3. Page 16, last line: Font size is large.

***Authors’ Response***

We are sorry for our careless mistake. We made adequate correction.

Again, thank you very much because the results of additional analysis you suggested have strengthened our conclusion and our manuscript has been improved very much.

[Note: HTML markup is below. Please do not edit.] Reviewers' comments:

Reviewer's Responses to Questions

Comments to the Author

1. If the authors have adequately addressed your comments raised in a previous round of review and you feel that this manuscript is now acceptable for publication, you may indicate that here to bypass the “Comments to the Author” section, enter your conﬂict of interest statement in the “Conﬁdential to Editor” section, and submit your "Accept" recommendation.

Reviewer #1: All comments have been addressed

Reviewer #2: All comments have been addressed

2. Is the manuscript technically sound, and do the data support the conclusions?

The manuscript must describe a technically sound piece of scientiﬁc research with data that supports the conclusions. Experiments must have been conducted rigorously, with appropriate controls, replication, and sample sizes. The conclusions must be drawn appropriately based on the data presented.

Reviewer #1: Yes

Reviewer #2: Yes

3. Has the statistical analysis been performed appropriately and rigorously?

Reviewer #1: Yes

Reviewer #2: Yes

4. Have the authors made all data underlying the ﬁndings in their manuscript fully available?

The [PLOS Data policy](http://www.plosone.org/static/policies.action#sharing) requires authors to make all data underlying the ﬁndings described in their manuscript fully available without restriction, with rare exception (please refer to the Data Availability Statement in the manuscript PDF ﬁle). The data should be provided as part of the manuscript or its supporting information, or deposited to a public repository. For example, in addition to summary statistics, the data points behind means, medians and variance measures should be available. If there are restrictions on publicly sharing data̶e.g. participant privacy or use of data from a third party̶those must be speciﬁed.

Reviewer #1: Yes

Reviewer #2: Yes

5. Is the manuscript presented in an intelligible fashion and written in standard English?

PLOS ONE does not copyedit accepted manuscripts, so the language in submitted articles must be clear, correct, and unambiguous. Any typographical or grammatical errors should be corrected at revision, so please note any speciﬁc errors here.

Reviewer #1: Yes

Reviewer #2: Yes

6. Review Comments to the Author

Please use the space provided to explain your answers to the questions above. You may also include additional comments for the author, including concerns about dual publication, research ethics, or publication ethics. (Please upload your review as an attachment if it exceeds 20,000 characters)

Reviewer #1: (No Response)

Reviewer #2: The paper is very solid and definitely publishable. They could consider including a recent paper of close to 900 followed 7 years showing peak VO2 after CR is the strongest predictor of survival ( Carbone Set al. EHJ-QCCO 2022; 8: 361-367) and a State of the Art discussing how cardiorespiratory fitness predicts survival following CR ( Tutor A et al. Prog Cardiovasc Dis 2022; 70: 2-7.)

***Authors’ Response***

Your suggestion is appreciated very much. Two manuscripts which you suggested were cited as new references #21 and #22, respectively. Also, the following sentences were added in the Discussion section.

Page 14/R2, Lines 176-178

“Recent studies have demonstrated that achieved peak VO_2_ after CR can serve as a predictor of long-term survival in patients with CVD [21,22].”

References

21.	Carbone S, Kim Y, Kachur S, Billingsley H, Kenyon J, De Schutter A, et al. Peak oxygen consumption achieved at the end of cardiac rehabilitation predicts long-term survival in patients with coronary heart disease. Eur Hear J - Qual Care Clin Outcomes. 2022;8: 361–367. doi:10.1093/ehjqcco/qcab032

22.	Tutor A, Lavie CJ, Kachur S, Dinshaw H, Milani R V. Impact of cardiorespiratory fitness on outcomes in cardiac rehabilitation. Prog Cardiovasc Dis. 2022;70: 2–7. doi:10.1016/j.pcad.2021.11.001

7. PLOS authors have the option to publish the peer review history of their article ([what does this mean?](https://journals.plos.org/plosone/s/editorial-and-peer-review-process#loc-peer-review-history)). If published, this will include your full peer review and any attached ﬁles.

If you choose “no”, your identity will remain anonymous but your review may still be made public.

Do you want your identity to be public for this peer review? For information about this choice, including consent withdrawal, please see our [Privacy Policy](https://www.plos.org/privacy-policy).

Reviewer #1: No

Reviewer #2: Yes: Carl " Chip" Lavie MD

[NOTE: If reviewer comments were submitted as an attachment file, they will be attached to this email and accessible via the submission site. Please log into your account, locate the manuscript record, and check for the action link "View Attachments". If this link does not appear, there are no attachment files.]

While revising your submission, please upload your figure files to the Preflight Analysis and Conversion Engine (PACE) digital diagnostic

tool, <https://pacev2.apexcovantage.com/>. PACE helps ensure that figures meet PLOS requirements. To use PACE, you must first register as a user. Registration is free. Then, login and navigate to the UPLOAD tab, where you will find detailed instructions on how to use the tool. If you encounter any issues or have any questions when using PACE, please email PLOS at [figures@plos.org](mailto:figures@plos.org). Please note that Supporting Information files do not need this step.

[*In compliance with data protection regulations, you may request that we remove your personal registration details at any time. (Remove my information/details). Please contact the publication oﬃce if you have any questions.*](https://www.editorialmanager.com/pone/login.asp?a=r)
