# Supplementary material for: Low serum brain-derived neurotrophic factor may predict poor response to cardiac rehabilitation in patients with cardiovascular disease
Source: PLoS One. 2024 Feb 6;19(2):e0298223. doi: 10.1371/journal.pone.0298223 (PMC10846715; doi:10.1371/journal.pone.0298223)
Supplement: Attachment — Submitted filename: Response to Reviewers R3.docx [file pone.0298223.s004.docx]

PONE-D-23-00760R2

Low serum Brain-Derived Neurotrophic Factor may predict poor response to cardiac rehabilitation in patients with cardiovascular disease

PLOS ONE

Dear Dr. Tsukada,

Thank you for submitting your manuscript to PLOS ONE. After careful consideration, we feel that it has merit but does not fully meet PLOS ONE’s publication criteria as it currently stands. Therefore, we invite you to submit a revised version of the manuscript that addresses the points raised during the review process.

Please submit your revised manuscript by Nov 30 2023 11:59PM. If you will need more time than this to complete your revisions, please reply to this message or contact the journal office at [plosone@plos.org](mailto:plosone@plos.org). When you're ready to submit your revision, log on to <https://www.editorialmanager.com/pone/> and select the 'Submissions Needing Revision' folder to locate your manuscript file.

Please include the following items when submitting your revised manuscript:

- A rebuttal letter that responds to each point raised by the academic editor and reviewer(s). You should upload this letter as a separate file labeled 'Response to Reviewers'.
- A marked-up copy of your manuscript that highlights changes made to the original version. You should upload this as a separate file labeled 'Revised Manuscript with Track Changes'.
- An unmarked version of your revised paper without tracked changes. You should upload this as a separate file labeled 'Manuscript'.

If you would like to make changes to your financial disclosure, please include your updated statement in your cover letter. Guidelines for resubmitting your figure files are available below the reviewer comments at the end of this letter.

If applicable, we recommend that you deposit your laboratory protocols in [protocols.io](http://protocols.io/) to enhance the reproducibility of your results. Protocols.io assigns your protocol its own identifier (DOI) so that it can be cited independently in the future. For instructions see: <https://journals.plos.org/plosone/s/submission-guidelines#loc-laboratory-protocols>. Additionally, PLOS ONE offers an option for publishing peer-reviewed Lab Protocol articles, which describe protocols hosted on [protocols.io](http://protocols.io/). Read more information on sharing protocols at <https://plos.org/protocols?utm_medium=editorial-email&utm_source=authorletters&utm_campaign=protocols>.

We look forward to receiving your revised manuscript.

Kind regards,

Yuichiro Nishida
Academic Editor
PLOS ONE

**Additional Editor Comments:**

You performed additional statistical analyses as requested by me. However, unfortunately, there are still a few severe concerns related to the additional analyses. I feel that your revision is insufficient.

#1. RE: The conclusion (described in the Abstract and the last paragraph of the Discussion section) is still mainly supported by the within-group comparison of AT and peak VO2. As I previously suggested, the conclusion must be based on the between-group comparison of the difference (%change) in the aerobic capacity indices.

***Authors’ response***

We thank you for pointing out that the revision of our conclusion in the Abstract and Discussion section was insufficient. According to your suggestion, we revised the Abstract, Results, and Discussion in the R3 manuscript.

In the Abstract

The original sentences describing our conclusions (page 3/R2, lines 39-41) were rewritten as follows, to read:

page 3/R3, lines 41-42

“Positive correlation with baseline BDNF levels was found for the CR-induced increases in peak VO2. It was suggested that CVD patients with low baseline BDNF levels may be poor responders to CR.”

In the Results section

The original sentence describing the results of ANCOVA for the comparison of age- and gender-adjusted %increase in peak VO2 (page 12/2R, line 157-page 13/2R, line 1) was changed as follows, to read:

page 12/R3, line 154-page 13/R3, line 157

“As shown in Figure 2, ANCOVA followed by post-hos analysis showed that the age- and gender-adjusted %increases in peak VO_2_ after 3-week CR were positively correlated with baseline BDNF levels (p=0.0239) and that High BDNF group had significantly higher %increase in peak VO_2_ than Low BDNF group (p=0.0197).”

In the Discussion section

The original sentence (page 13/R2, line 168-page 14, line 170) was revised as follows, to read:

page 14/R3, lines 174-177

“After adjustment for age- and gender, a positive correlation with baseline BDNF levels was found for %increases in peak VO_2_, but not in AT, after 3-week CR, and Low BDNF group had significantly lower %increase in peak VO_2_ than High BDNF group.”

In the Conclusion section

The original sentences (page 19/R2, lines 244-247) were revised as follows, to read:

page 20/R3, lines 262-page 21, line 266

“In conclusion, low baseline BDNF levels were associated with malnutrition in CVD patients. And, a positive correlation with baseline BDNF levels was shown for the CR-induced increases in peak VO_2_. It was suggested that CVD patients with low baseline BDNF levels may be poor responders to CR during the subacute phase. Therefore, BDNF can be a helpful biomarker for creating a tailored, optimized exercise program in CVD patients.”

#2. RE: In the Methods in the Abstract, there is no explanation of the statistical analysis mainly used. A description of the between-group comparison of the difference (%change) in aerobic capacity indices using ANCOVA is missing.

***Authors’ response***

According to your suggestion, the original sentences (page 2/R2, lines 35-38) were changed, as follows, to read:

In the Abstract

page 2/R3, lines 35-page 3/R3, line 38

“Analysis of covariance followed by post-hos analysis using Turkey’s HSD test revealed that age- and gender-adjusted %increases in peak VO2 after 3-week CR was positively correlated with baseline BDNF levels (p=0.0239) and Low BDNF group showed significantly lower %increase in peak VO2 than High BDNF group (p=0.0197).”

#3. RE: Please clearly mention which statistical analysis is mainly used to make a conclusion in the Statistical Analysis section. For instance, “Analysis of covariance (ANCOVA) followed by post-hoc analysis using Turkey’s HSD test was conducted to compare age- and gender-adjusted %increase in AT and peak VO2 after 3-week CR, as a main statistical analysis of the present study”.

***Authors’ response***

As you suggested, the original sentence (page 9/2R, lines 128-130) was rewritten as follows, to read:

In the Method section

Page 9/R3, lines 128-130

“Analysis of covariance (ANCOVA) followed by post-hoc analysis using Turkey’s HSD test was conducted to compare age- and gender-adjusted %increases in AT and peak VO_2_ after 3-week CR, as a main statistical analysis of the present study.”

#4. RE: In the Results section, you mentioned that “However, age- and gender-adjusted %increase in AT did not differ among the three groups”. Please show exact P values for these differences. Did age- and gender-adjusted %increase in AT not show a similar tendency as the peak VO2?

***Authors’ response***

The exact p value was added as follows, to read:

In the Abstract

Page 3/R3, Lines 38-39

“Significant association was not found between baseline BDNF and %increase in AT (p=0.1379).”

In the Results section

Page 13/R3, line 157-159

“In contrast, significant association with baseline BDNF was not found for %increase in AT after 3-week CR (p=0.1379).”

To replay your question, we performed an additional analysis comparing the age- and gender-adjusted %increases in AT between Low BDNF group and Middle+High BDNF group. The result was added in the Result section as follows, to read:

Page 13/R3, lines 159-162

“When the age- and gender-adjusted %increases in AT were compared between Low BDNF group and Middle+High BDNF group, the adjusted %increase in AT tended to be higher in Middle+High BDNF group (9.53±2.16%) than in Low BDNF group (2.51±3.17%) although the difference was not significant (p=0.0689).”

#5. RE: If there is not that tendency, please touch on the potential reason why the significant effect was seen only in the peak VO2 in the Discussion section.

***Authors’ response***

As you suggested, the discussion on this issue was added in the R3 manuscript:

In the limitation section of the Discussion

page 19/R3, lines 248-page 20/R3, line 258

“From the present study, it remains unknown why significant correlation with baseline BDNF was found for the CR-induced increase in peak VO_2_, but not for that in AT. A possible explanation of this discrepancy could be that AT changes are less clearly manifested than peak VO_2_ changes because the effects of BDNF levels on AT would involve more complex and multifactorial mechanism compared with the effects on peak VO_2_. Namely, AT serves as a comprehensive measure of exercise tolerance including the respiratory, circulatory, and metabolic processes associated with exercise, whereas peak VO_2_ is an indicator of exercise tolerance based primarily on oxygen delivery capacity [35]. Another possibility is that the 3-week CR period during hospitalization may be too short for baseline BDNF levels to affect AT changes. Molecular and physiological mechanisms whereby BDNF affects peak VO_2_ and AT should be determined in future studies.”

And, new reference was added as reference #35 in the R3 manuscript.

35. Poole DC, Rossiter HB, Brooks GA, Gladden LB. The anaerobic threshold: 50+ years of controversy. J Physiol. 2021;599: 737–767. doi:10.1113/JP279963.

[Note: HTML markup is below. Please do not edit.]

[NOTE: If reviewer comments were submitted as an attachment file, they will be attached to this email and accessible via the submission site. Please log into your account, locate the manuscript record, and check for the action link "View Attachments". If this link does not appear, there are no attachment files.]

While revising your submission, please upload your figure files to the Preflight Analysis and Conversion Engine (PACE) digital diagnostic tool, <https://pacev2.apexcovantage.com/>. PACE helps ensure that figures meet PLOS requirements. To use PACE, you must first register as a user. Registration is free. Then, login and navigate to the UPLOAD tab, where you will find detailed instructions on how to use the tool. If you encounter any issues or have any questions when using PACE, please email PLOS at [figures@plos.org](mailto:figures@plos.org). Please note that Supporting Information files do not need this step.

*In compliance with data protection regulations, you may request that we remove your personal registration details at any time.* [*(Remove my information/details)*](https://www.editorialmanager.com/pone/login.asp?a=r)*. Please contact the publication office if you have any questions.*
