# Supplementary material for: Low serum brain-derived neurotrophic factor may predict poor response to cardiac rehabilitation in patients with cardiovascular disease
Source: PLoS One. 2024 Feb 6;19(2):e0298223. doi: 10.1371/journal.pone.0298223 (PMC10846715; doi:10.1371/journal.pone.0298223)
Supplement: Attachment — Submitted filename: Response to Reviewrs R4.docx [file pone.0298223.s005.docx]

PONE-D-23-00760R3

Low serum Brain-Derived Neurotrophic Factor may predict poor response to cardiac rehabilitation in patients with cardiovascular disease

PLOS ONE

Dear Dr. Tsukada,

Thank you for submitting your manuscript to PLOS ONE. After careful consideration, we feel that it has merit but does not fully meet PLOS ONE’s publication criteria as it currently stands. Therefore, we invite you to submit a revised version of the manuscript that addresses the points raised during the review process.

Please submit your revised manuscript by Jan 19 2024 11:59PM. If you will need more time than this to complete your revisions, please reply to this message or contact the journal office at [plosone@plos.org](mailto:plosone@plos.org). When you're ready to submit your revision, log on to <https://www.editorialmanager.com/pone/> and select the 'Submissions Needing Revision' folder to locate your manuscript file.

Please include the following items when submitting your revised manuscript:

- A rebuttal letter that responds to each point raised by the academic editor and reviewer(s). You should upload this letter as a separate file labeled 'Response to Reviewers'.
- A marked-up copy of your manuscript that highlights changes made to the original version. You should upload this as a separate file labeled 'Revised Manuscript with Track Changes'.
- An unmarked version of your revised paper without tracked changes. You should upload this as a separate file labeled 'Manuscript'.

If you would like to make changes to your financial disclosure, please include your updated statement in your cover letter. Guidelines for resubmitting your figure files are available below the reviewer comments at the end of this letter.

If applicable, we recommend that you deposit your laboratory protocols in [protocols.io](http://protocols.io) to enhance the reproducibility of your results. Protocols.io assigns your protocol its own identifier (DOI) so that it can be cited independently in the future. For instructions see: <https://journals.plos.org/plosone/s/submission-guidelines#loc-laboratory-protocols>. Additionally, PLOS ONE offers an option for publishing peer-reviewed Lab Protocol articles, which describe protocols hosted on [protocols.io](http://protocols.io). Read more information on sharing protocols at <https://plos.org/protocols?utm_medium=editorial-email&utm_source=authorletters&utm_campaign=protocols>.

We look forward to receiving your revised manuscript.

Kind regards,

Yuichiro Nishida
Academic Editor
PLOS ONE

**Journal Requirements:**

RE: *Please review your reference list to ensure that it is complete and correct. If you have cited papers that have been retracted, please include the rationale for doing so in the manuscript text, or remove these references and replace them with relevant current references. Any changes to the reference list should be mentioned in the rebuttal letter that accompanies your revised manuscript. If you need to cite a retracted article, indicate the article’s retracted status in the References list and also include a citation and full reference for the retraction notice*.

***Author’s response***

First of all, we have ensured that we have not cited any papers that have been retracted.

Next, after carefully checking our reference list, we found the duplication of the references #13 and #29 in the R3 manuscript. Thus, we have deleted the reference #29 and have renumbered all references. Now, we have ensured that the reference list is complete and correct in the R4 manuscript. We apologize for our careless mistake.

**Additional Editor Comments:**

#1. RE: *The description regarding the association analysis (as one of the main statistical analyses in the present study to support the conclusion) is completely missing. Did you analyze the multivariate association of baseline BDNF (as an independent variable) with the % increases in AT or peak VO2 (as dependent variables) with adjustment for age and gender? Is the baseline BDNF (as an independent variable) treated as a continuous (mg/ml) or categorical [low, middle, high] variable? Similarly, is age (as a covariate) treated as a continuous (years) or categorical variable (another covariate gender should be treated as a categorical variable)? This important information should be clearly written in the manuscript (in the Methods in the Abstract and the Statistical Analysis in the Methods section). I have recognized that the description regarding the other main statistical analysis ANCOVA is also missing in the Methods in the Abstract.*

***Author’s response***

We appreciate your suggestion in the decision letter to PONE-D-23-00760R1, dated on 6 Jun 2023, that “*to adjust for potential confounding factors, an analysis of covariance (ANCOVA) may be used to calculate the adjusted means (and 95% confidence intervals) of the changes in AT and Peak VO2 in the three groups.*” and “*you should use multivariate analysis (e.g., ANCOVA) to adjust for at least age and sex as covariates.*” Thus, we conducted ANCOVA for multivariate analysis as the main statistical analysis in the R2 and R3 manuscripts. But, as you pointed out, we are sorry that we failed to describe the important information regarding the ANCOVA analysis in these versions of our manuscript. Thus, in the R4 manuscript, the following changes were done as follows, to read:

In the Statistical Analysis in the Methods section,

The original sentence: “Analysis of covariance (ANCOVA) followed by post-hoc analysis using Tukey’s HSD test was conducted to compare age- and gender-adjusted %increases in AT and peak VO_2_ after 3-week CR, as a main statistical analysis of the present study.” (page 9/R3, lines 128-130) was changed as follows, to read:

page 9/R4, lines 130-136

“Analysis of covariance (ANCOVA) was conducted to assess the multivariate associations between baseline BDNF levels (as independent variable) and %increases in AT and peak VO_2_ after 3-week CR (as dependent variables) after adjustment for age and gender (as covariates), as a main statistical analysis of the present study. And, ANCOVA followed by post-hoc analysis using Tukey’s HSD test was used to compare age- and gender-adjusted %increases in AT and peak VO_2_ after 3-week CR. Baseline BDNF levels were treated as a categorical data based on BDNF tertiles. Age and gender were treated as a continuous and categorical data, respectively.”

In the Abstract,

New sentences were added to the Method section of the Abstract. Please note that total word count for the Abstract is limitation to be 300 words or less. Thus, we appreciate very much if you understand that it is not possible to provide more detailed information in the Abstract.

Page 2/R4, lines 32- 36

“Analysis of covariance (ANCOVA) followed by post-hoc analysis using Tukey’s HSD test was conducted to assess the multivariate associations between baseline BDNF levels categorized by BDNF tertiles (as independent variable) and %increases in AT and peak VO_2_ after 3-week CR (as dependent variables) after adjustment for age and gender (as covariates), as a main statistical analysis of the present study.”

And, the original words: “Analysis of covariance (ANCOVA) followed by post-hoc analysis using Tukey’s HSD test” (page 2/R3, lines 35-36) was changed to:

Page 3/R4, line 39

“ANCOVA followed by post-hoc analysis”.

After the above revisions, the total word count for the Abstract is 300 words.

**#2. RE: *The expression regarding the main result “correlation with baseline BDNF levels was found for the CR-induced increases in peak VO2” is somewhat strange. Please change the expression from “correlation with baseline BDNF levels was found for the CR-induced increases in peak VO2” to “ (significant) association between baseline BDNF levels and CR-induced increase in peak VO2 was found (or [significant] association of baseline BDNF levels with CR-induced increase in peak VO2 was found)” throughout the manuscript.***

***Author’s response***

Thank you for your comment. We have rewritten the sentences, which you pointed out, throughout the manuscript, according to your suggestion.

In the Abstract,

Page 3/R4, line 40

The original word: “correlated” (page 2/R3, line 37) was replaced to “associated”.

The original sentence: “Positive correlation with baseline BDNF levels was found for the CR-induced increases in peak VO_2_.” (page 3/R4, line 41) was changed as follows, to read:

Page 3/R4, lines 44-46

“A positive association between baseline BDNF levels and CR-induced increases in peak VO_2_ was found.”

In the Results,

Page 12/R4, line 164

The original word: “correlated” (page 13/R3, line 156) was replaced to “associated”.

The original sentence: “In contrast, significant association with baseline BDNF was not found for %increase in AT after 3-week CR was not found (p=0.1379).” (page 13/R3, lines 157-158) was changed as follows, to read:

Page 12/R4, line 165-page 13/R4, line 167

“In contrast, significant association between baseline BDNF and %increase in AT after 3-week CR was not found (p=0.1379).”

In the Discussion,

The original sentence: “a positive correlation with baseline BDNF levels was found for %increases in peak VO_2_, but not in AT, after 3-week CR,” (page 14/R3, lines 175-176) was rewritten as follows, to read:

Page 14/R4, line 183-184

“a positive association between baseline BDNF levels and %increases in peak VO_2_, but not in AT, was found after 3-week CR,”

The original sentence: “From the present study, it remains unknown why significant correlation with baseline BDNF was found for the CR-induced increase in peak VO_2_, but not for that in AT.” (page 19/R3, line 248-249) was changed as follows, to read:

Page 19/R4, lines 256-257

“From the present study, it remains unknown why significant association between baseline BDNF and the CR-induced increase in peak VO_2_, but not in AT, was found.”.

The original sentence: “a positive correlation with baseline BDNF levels was shown for the CR-induced increases in peak VO_2_.” (page 20/R3, line 263-264) was rewritten as follows, to read:

Page 20/R4, lines 271-272

“a positive association between baseline BDNF levels and the CR-induced increases in peak VO_2_ was shown.”.

Dear Editor, we are very sorry for our careless mistake. We found an incomplete sentence in the Discussion section in the R3 manuscript and have corrected it in the R4 manuscript.

In the Discussion,

The original sentence: “From this study, the association between serum BDNF and skeletal muscle strength in CVD patients.” (page 17/R3, lines 221-222) was incomplete. Thus, the following change was done in the R4 manuscript.

Page 17/R4, lines 229-230

“From this study, the association between serum BDNF and skeletal muscle strength remains undetermined in CVD patients.”

[Note: HTML markup is below. Please do not edit.]


[NOTE: If reviewer comments were submitted as an attachment file, they will be attached to this email and accessible via the submission site. Please log into your account, locate the manuscript record, and check for the action link "View Attachments". If this link does not appear, there are no attachment files.]

While revising your submission, please upload your figure files to the Preflight Analysis and Conversion Engine (PACE) digital diagnostic tool, <https://pacev2.apexcovantage.com/>. PACE helps ensure that figures meet PLOS requirements. To use PACE, you must first register as a user. Registration is free. Then, login and navigate to the UPLOAD tab, where you will find detailed instructions on how to use the tool. If you encounter any issues or have any questions when using PACE, please email PLOS at [figures@plos.org](mailto:figures@plos.org). Please note that Supporting Information files do not need this step.
